# Perturbation to the nitrogen cycle during rapid Early Eocene global warming

**DOI:** 10.1038/s41467-018-05486-w

**Published:** 2018-08-09

**Authors:** Christopher K. Junium, Alexander J. Dickson, Benjamin T. Uveges

**Affiliations:** 1 Department of Earth Sciences, Syracuse University, Syracuse, NY 13244 USA; 2 Department of Earth Sciences, Royal Holloway University of London, Egham, Surrey TW20 0EX UK

## Abstract

The degree to which ocean deoxygenation will alter the function of marine communities remains unclear but may be best constrained by detailed study of intervals of rapid warming in the geologic past. The Paleocene–Eocene Thermal Maximum (PETM) was an interval of rapid warming that was the result of increasing contents of greenhouse gases in the atmosphere that had wide ranging effects on ecosystems globally. Here, we present stable nitrogen isotope data from the Eastern Peri-Tethys Ocean that record a significant transition in the nitrogen cycle. At the initiation of the PETM, the nitrogen isotopic composition of sediments decreased by ~6‰ to as low as −3.4‰, signaling reorganization of the marine nitrogen cycle. Warming, changes in ocean circulation, and deoxygenation caused a transition to nitrogen cycle to conditions that were most similar to those experienced during Oceanic Anoxic Events of the Mesozoic.

## Abstract (web summary)

Studying the PETM, a past period of rapid warming ~56 Ma, could provide insights into ecosystem response under future warming conditions. Here, the authors present stable nitrogen isotope data that reveal a dramatic change in the marine nitrogen cycle and the emergence of anoxic conditions.

## Introduction

Productivity in the marine environment is ultimately controlled by the cycling of nitrogen, phosphorus, iron, and other bio-limiting trace elements. The degree to which nutrient cycling and marine productivity will be affected by future warming scenarios remains unclear, but the effects can be revealed through the careful investigation of past intervals of rapid warming^1^. One aspect of future change that has received attention is ocean deoxygenation because of its potential for serious impacts to coastal ecosystems^2,3^. Recent efforts aimed at quantifying the degree of O_2_ change in the ocean over the last 50 years have revealed significant decreases in dissolved O_2_ concentrations, a trend that is expected to continue^4^. Predicting how the nitrogen cycle might evolve in response to future oceanic redox changes is an important goal, and can be investigated from the study of rapid environmental change events in the geologic past.

The Paleocene–Eocene Thermal Maximum (PETM; ~56 Ma) was the result of at least 1500 GT of carbon and perhaps as much as 10,000 GT added to the earth–ocean–atmosphere system over less than 10 kyr^5–8^. Carbon addition caused rapid warming of Earth’s surface by ~5 °C globally^9^. Beyond warming, the environmental and biological consequences of rapid carbon cycle change^10^ have made the PETM the focus of many studies that utilize geological and paleontological records as analogues for the potential impact of future warming on marine environments. How the PETM affected nutrient cycling remains an open, and potentially important question. In an effort to help illuminate this issue, we present highly-resolved nitrogen isotope data in deposits spanning the PETM, from the northeast margin of the Tethys Ocean. The new data demonstrate that a dramatic change in the nitrogen cycle occurred during the PETM to conditions that may be similar to episodes of expanded marine anoxia known as Oceanic Anoxic Events (OAEs) that punctuated the Mesozoic greenhouse.

## Results

### Geologic setting

**Fig. 1:**
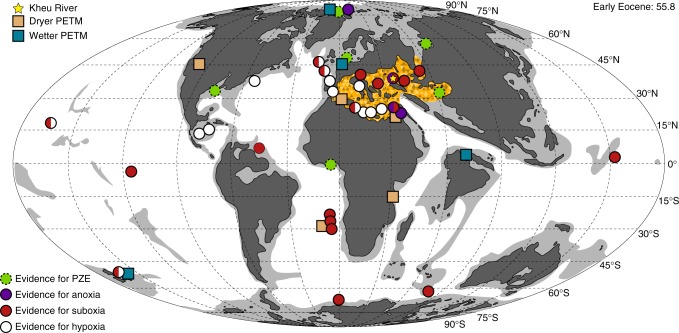
Paleogeographic map of the PETM. Map adapted from plate tectonic reconstructions by Scotese^66^ with relevant data on redox and hydrologic indicators at PETM sites. Deoxygenation and hydrologic data from Carmichael et al.^20^ and references therein. Orange shaded region shows the extent of strata that displayed TOC enrichment from background during the PETM in the Tethys^11,13,16,67,68^

The Kheu River site is located in the Karbardino-Balkaria Republic, Russian Federation (Fig. 1). The succession was located on the northern part of the wide, shallow Peri-Tethys seaway, which was dramatically flooded starting in the Late Paleocene^11,12^. The base of the section is composed of calcareous mudrock of latest Paleocene age, as determined from nannofossil biostratigraphy. This unit contains a depositional hiatus that represents a flooding surface during the late-Paleocene transgression in the northern Peri-Tethys. The timing of this flooding surface is diachronous: at more northerly locations (Khazakstan), it corresponds to the base of an organic matter-rich mudrock (sapropel) level; in the southern Peri-Tethys, in the region of Kheu River, the transgressive event occurs below the sapropel level. These differences indicate sedimentary onlap in a northerly direction^11^.

Calcareous mudrocks at Kheu River are conformably overlain by a thin layer of organic-lean claystone that abruptly transitions into a ~1 m thick organic matter-rich mudrock. This unit contains organic matter concentrations between 2 and 9 wt% total organic carbon (TOC), in two distinct TOC peaks. The base of the sapropel horizon is tied to the Paleocene–Eocene boundary by the appearance of the dinocyst *Apectodinium augustum*^12,13^, which is ubiquitous in deposits of this age (and occurs at the base of the PETM event)^14^. The initial 10 cm of the sapropel is finely laminated, above which the sapropel is occasionally bioturbated by *Chondrites*, an ichnofossil consistent with intermittent low-oxygen conditions at the benthic boundary layer^13,15^. The age of this unit also falls within nannofossil zones NP9b and 10^13,15^, which occur within the PETM carbon-isotope excursion at the Paleocene–Eocene GSSP at Dababiya Quarry, Egypt^16^. The sapropel horizon at Kheu River grades conformably into organic-lean calcareous mudstones, and eventually into siliceous mudstones at the top of the sedimentary exposure.

Paleo-depth indicators suggest a neritic depth (100–200 m) assignment for the Kheu River succession. This assignment is determined on the basis of the benthic fauna, including foraminifera with neritic depth habitats, large ostracods (requiring proximity to the photic zone), and dinocyst assemblages^15^. The Kheu River sapropel horizon constitutes an organic-rich unit that can be litho-, chemo-, and bio-stratigraphically correlated to a large number of sites extending throughout the northern Peri-Tethys region, and which is consistent with a dramatic restructuring of the nutrient and redox regime to this region during the PETM.

### Geochemical data

**Fig. 2:**
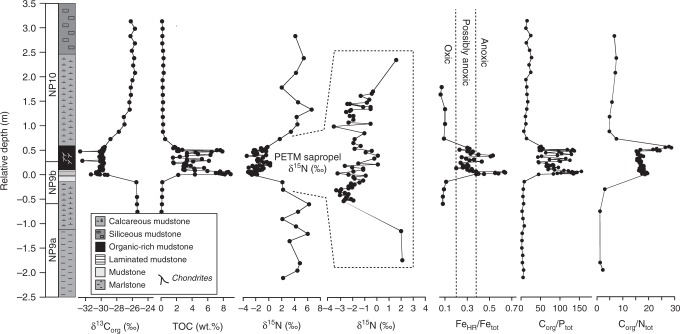
Geochemical data from the Kheu River section. Primary variability in the nitrogen data reflect the facies shifts that correspond to the transitions into and out of the interval of sapropel deposition. Nitrogen-isotope data between the calcareous shales and the interbedded sapropel do not overlap, and underscore the differences in sapropel and non-sapropel geochemical conditions (Fig. 3)

**Fig. 3:**
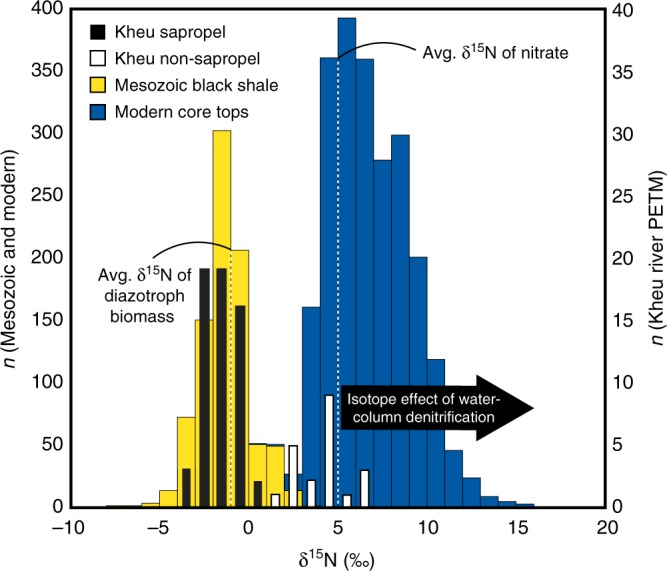
Histogram of relevant nitrogen isotope data. Sedimentary nitrogen isotope data are from Kheu River, Mesozoic black shales, and modern core tops^38,41,44,48,69–73^ in 1‰ bins

Nitrogen isotope data range from +6.2 to +2.0‰ in the calcareous mudstones deposited prior to and after the PETM, and range from 0.2 to −3.4‰ within the PETM sapropel (Figs. 2 and 3). C/N ratios (C_org_/N_tot_) range from 16.0 to 28.7 within the sapropel and 1.2 to 7.5 in the calcareous mudstones. Fe-speciation measurements [previously reported in Dickson et al.^13^] demonstrate highly reactive Fe to total Fe ratios (Fe_HR_/Fe_T_) above and below the sapropel bed that are <0.1, consistent with deposition under oxidizing conditions. Within the sapropel, Fe_HR_/Fe_tot_ is greater than 0.38 at the top and base of the sapropel bed, indicating predominantly anoxic depositional conditions. At the initiation of sapropel deposition the ratio of pyrite-Fe to highly reactive Fe (Fe_PY_/Fe_HR_) is >0.8, suggesting a brief episode of euxinia (sulfidic water column conditions). Gross variability in the geochemical data reflect the facies shifts that correspond to the transitions into and out of the interval of sapropel deposition during the PETM (Fig. 1). Nitrogen isotope data between the calcareous shales and the interbedded sapropel do not overlap (Fig. 3), and underscore the differences between sapropel and non-sapropel geochemical conditions. Comparison of geochemical parameters limited to within the sapropel bed reveals significant relationships between δ^15^N and C_org_/P_tot_ and C_org_/Al_tot_ (C_org_/P_tot_, *r*^2^ = 0.42, *p* < 0.01; C_org_/Al_tot_, *r*^2^ = 0.44, *p* < 0.01; Supplementary Fig. 1).

## Discussion

The PETM does not feature expansive deposition of organic matter-rich black shales nor is there evidence for severe deoxygenation in open marine settings in a manner that is directly analogous to the Mesozoic OAEs. Whilst there is substantial evidence that O_2_ content decreased in the open ocean during the PETM^17–19^, severe O_2_ reductions were focused within the Arctic and Tethyan realms^20–24^ but have been noted in other locations^25^. New geochemical proxies that are sensitive to differing changes in redox state paint a more nuanced picture of the degree of deoxygenation during the PETM. Iodine geochemistry indicates that O_2_ concentrations at intermediate water depths in open ocean settings (1–2 km) decreased, signaling the expansion of oxygen minimum zones (OMZs) during the PETM^19^. Molybdenum stable isotopes suggest that the proportion of euxinic waters, those containing free sulfide, also expanded during the PETM^23^. The biomarkers for phototrophic sulfide-oxidizing, green sulfur bacteria have been reported for shelf environments and provide direct evidence for euxinia in relatively shallow shelf sequences^22,25–27^.

**Fig. 4:**
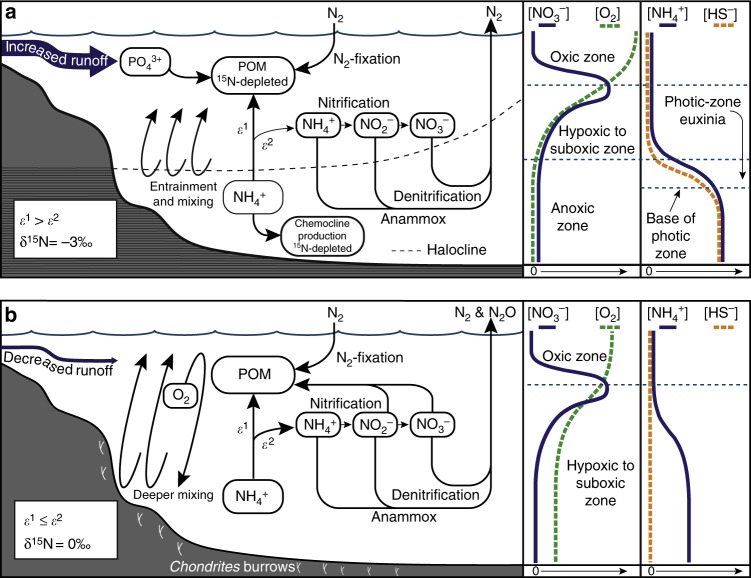
Schematic diagram of the nitrogen cycle during the PETM at Kheu River. **a** The nitrogen cycle during the initial and late stages of the PETM when the water column was predominantly anoxic. Nitrogen isotope depletion was driven by mixing and entrainment of ammonium from below the chemocline into the lower photic zone. In this situation, high ammonium concentrations allowed for the expression of an uptake fractionation (*ε*^1^) by primary producers^41^. Less efficient utilization (*ε*^2^) of ammonia by nitrifying archaea and bacteria, and its subsequent reduction to N_2_ via denitrification and anammox serve as a sink for residual ^15^N-enriched ammonium. **b** During the middle interval of the PETM at Kheu River, deeper mixing allowed for invasion of O_2_ to the benthic boundary layer, development of suboxic/hypoxic conditions that may have allowed for the production of N_2_O and relative ^15^N-enrichment

The expansion of euxinia^23^ and increased organic carbon burial in coastal environments^28^ highlight the interplay between the flux of weathering-derived nutrients and the hydrography of shelf systems. Bursts of nutrient-rich fresh water into coastal systems in an intensifying hydroclimate^29^ might have been sufficient to drive short-term reductions in water column oxygen content as organic matter export increased. However, the more pronounced anoxia that occurred over wide regions of Eastern Peri-Tethys during the PETM^11^ (Fig. 1) required longer-term changes in the style of regional circulation and/or nutrient availability. Gavrilov et al.^11^ hypothesize that the combined effect of transgression and an increased flux of riverine-derived nutrients were the likely mechanisms that set the stage for sapropel deposition in the Eastern Tethys. Epicontinental basins similar to the Eastern Tethys, such as the Laurentian basins of the Late Devonian^30^ and Cretaceous Western Interior Seaway^31^ maintained estuarine modes of circulation that fostered periods of black shale deposition. During the PETM in the Eastern Peri-Tethys, a regionally wetter hydroclimate^20^ coupled with transgression might have supported an estuarine circulation where less saline surface waters limited ventilation of more saline deepwaters. Despite the development of halostratification, estuarine circulation regimes are by no means stagnant. Rather, surface waters entrain and mix with anoxic, nutrient-rich deep waters while maintaining a dynamic chemostratification (Fig. 4).

Basinal anoxia, and euxinia in particular, enhance the accumulation of ammonium and phosphate derived from degraded organic matter within the anoxic region of the water column^32^. Significant increases in C_org_/P_tot_ ratios in the Kheu River (Fig. 2) section^13^ are consistent with this liberation of P from organic matter and sedimentary mineral phases^33^. Shallowing of the chemocline to an extent that allowed for expansion of photic zone euxinia during the PETM at Kheu River^12^ suggests that high nutrient concentrations were available to photoautotrophs as well as chemocline dwellers. Despite the extremes of anoxia experienced at Kheu River, evidence for bioturbation within the sapropel level indicates that there were significant oscillations in redox state. In this relatively shallow environment, relaxation of stratification and oxidation of the benthic boundary layer would have been accompanied by mixing of deep water nutrients to the surface, stimulating productivity. Here, we consider how the nitrogen isotopic record from Kheu River can illuminate the interaction between local nutrient dynamics and redox state, and serve as a point of perspective to more thoroughly consider the extent of nitrogen cycle perturbation during the PETM.

Nitrogen isotopes of organic matter from marine sediments provide a means for disentangling nitrogen cycle processes^34^. In particular, nitrogen isotopes are sensitive to changes in local water column redox state. The isotopic composition and reservoir size of dissolved inorganic nitrogen (DIN: NO_3_^−^, NO_2_^−^, and NH_4_^+^) reflect the relative influences of the principal nitrogen cycle processes. Biological nitrogen fixation (diazotrophy) serves as the primary input of new nitrogen to the ocean. The organic matter produced by diazotrophs has an isotopic composition that is pinned to the δ^15^N of the atmosphere with a minor fractionation that results in organic matter that is approximately −1‰^35^. Anaerobic respiratory processes that reduce nitrate and nitrite (NO_3_^−^ and NO_2_^−^) to N_2_ or N_2_O, occur in the low-O_2_ regions of the water column or within sediment pore waters, and constitute the most important sinks for nutrient nitrogen^36^. The biological isotope effect (*ε*) of nitrate reduction, inclusive of both denitrification and anaerobic ammonium oxidation (anammox) processes, is significant (*ε* = 10–30), and results in ^15^N-enrichment of the residual nitrate, in some cases to as high as +15‰^34,37^. The relative balance between diazotrophy and water column nitrate reduction is integrated by the average δ^15^N of deep water NO_3_^−^ in the modern ocean which is ~ +5‰^37^.

The water column nitrogen cycle signals imparted on DIN can be reliably traced in the δ^15^N of modern core top sedimentary organic matter. However, demonstrating that nitrogen isotope signals in sedimentary organic matter are reflective of water column processes has been an important hurdle in deep time paleoceanography. The δ^15^N of bulk sediments from modern environments, particularly under reducing conditions, preserve the isotopic composition of water column particulate biomass and sub-euphotic zone DIN^38–40^. In more ancient sedimentary sequences, bulk δ^15^N has been shown to be a faithful recorder of water column processes where thermal maturities are low. Further confirmation of the fidelity of bulk δ^15^N in ancient organic-matter-rich sediments has also been demonstrated through kerogen and compound-specific δ^15^N analyses of chlorophyll *a*-derived porphyrins. These studies demonstrate that even anomalously low nitrogen isotopic signatures in Cretaceous black shales reflect the δ^15^N of oxygenic photoautotrophic biomass, and are similar to those we observe in the Kheu river section^41–43^ (Fig. 3).

In consideration of the Kheu River nitrogen isotope record, it is unlikely that oxidation, organic matter mixing, or thermal maturity played a strong role in controlling the δ^15^N signatures during sapropel deposition. The organic geochemistry of the Kheu sapropel suggests that organic nitrogen was thermally immature and derived from a predominantly marine source of organic matter^13,15^. Terrestrially-derived organic matter is present throughout the section but contributed a relatively minor proportion of the total organic-matter^13,15^. The relationship between N (wt%) and TOC (wt%) within the sapropel is very strong (*R*^2^ = 0.97; *p* ⪡ 0.01) (Supplementary Fig. 2), and suggests that organic nitrogen is dominated by one source, without significant influence from nitrogen of detrital origin or other sources with significantly different C/N ratios. The elevated C/N ratios that we observe in the Kheu sapropel are typical of Phanerozoic black shales and sapropels, and are attributed to enhanced preservation of organic carbon, and relative loss of ammonium–N from sediments due to a limitation in the sorptive capacity of mineral surfaces^44,45^.

Prior to and following the PETM, oxic conditions prevailed during deposition of the calcareous mudrocks under and overlying the sapropel (Fig. 2). The relatively enriched δ^15^N (+1.7 to +6.6‰) in the calcareous mudrocks are typical of modern shelf settings that have oxic water columns^38^ (Fig. 3). Oxidizing conditions can be conducive to the preferential degradation of organic nitrogen, and alteration of δ^15^N may have occurred. Detailed investigations of the effect of degradation of organic δ^15^N in oxic sediments have demonstrated an increase in the order of 1‰ in organic nitrogen^45^. Despite the range of potential issues, the total effect on bulk δ^15^N is limited^40,46^. In the Holocene of the eastern Mediterranean, δ^15^N oscillations of up to 8‰ have been reported between sapropels and organic lean marls (wt% TOC < 0.25%), a signal that has been confirmed by compound-specific δ^15^N analyses of chlorophyll derivatives^47^. This similarity suggests that even in the deep eastern basin of the Mediterranean (depth > 1000 m) the oxic degradation of organic nitrogen had an inconsequential effect on the δ^15^N of bulk sediments. Accordingly, the δ^15^N of bulk sediments from the calcareous claystones of the Kheu River section are likely to be reasonable approximations of primary organic material.

At the onset of the PETM, the sharp decrease in δ^15^N was maintained for the duration of sapropel deposition and the PETM carbon isotope excursion (average = −1.64‰; 1*σ* = 0.88 *n* = 59). The δ^15^N minimum of −3.6‰ and distribution of δ^15^N data are strikingly similar to black shales deposited during Mesozoic OAEs^48^ (Fig. 3). The few nitrogen isotope records from the PETM where organic matter is present in sufficient quantities to be certain of an autochthonous source^49^ are limited to sites from the southern Tethys and the Arctic Basin where bulk δ^15^N is not lower than 0‰^8,50^. Nitrogen isotopic compositions that are significantly less than 0‰ in marine settings are largely unique to organic matter-rich sediments and mudrocks from Mediterranean sapropels and Mesozoic black shales (Fig. 3). This association suggests that the processes that controlled the nitrogen cycle during the PETM in the Eastern Tethys and during Phanerozoic OAEs may have shared similar biogeochemical mechanisms.

Nitrogen isotopic compositions that are significantly less than the nitrogen fixation end member (δ^15^N < −1‰) require a unique set of circumstances. The favored explanation for this degree of ^15^N-depletion relies upon the mixing of ammonium into the photic zone in excess of biochemical need, allowing for expression of the uptake fractionation by phytoplankton during ammonium assimilation^41^. The problem with ^15^N-depletion to this extent is that it requires a sink for the residual ^15^N-enriched ammonium that is in excess after assimilation. In the absence of this sink, complete utilization of ammonium would result in biomass δ^15^N equivalent to the δ^15^N of the ammonium available to phytoplankton.

Higgins et al.^41^ proposed a biochemical mechanism that hinges on the isotopic partitioning of the ammonium between phytoplankton and ammonia-oxidizing bacteria and archaea on the basis of differences in the magnitude of biological fractionation (*ε*) and rate of uptake (*φ*) between the two communities. When *ε* for ammonium assimilation by phytoplankton is greater than that of ammonia oxidation, the δ^15^N of sinking organic matter is lower than the δ^15^N of ammonium (Fig. 4). The fate of the residual oxidized ammonium (nitrate and nitrite) is key to this model: it must be reduced by denitrifiers and anammox bacteria and lost as N_2_ or N_2_O. Anammox and denitrification served as a terminal sink for the residual, relatively ^15^N-enriched nitrate and nitrite. The presence of ammonium can also limit the expression of the genes that encode for the membrane transport and assimilatory reduction of nitrate by phytoplankton^51^. Repression of nitrate assimilation would further favor the utilization of ammonium when available to oxygenic photoautotrophs, leaving nitrate and nitrite available for dissimilatory reduction by denitrifiers and anammox bacteria.

Accumulation of an ammonium reservoir is a precondition for the ^15^N-depletion that we observe in the Kheu sapropel. The geologic and geochemical evidence for persistent nutrient trapping and ammonium-rich deep waters at Kheu River is strong. Modern anoxic marine basins where ammonium concentrations are >5 μM are accompanied by measurable concentrations of sulfide^52,53^. Biomarker data indicating photic zone euxinia in the Eastern Peri-Tethys^26^ suggest that sulfide was at least episodically present within the photic zone. The δ^15^N minimum in the lowermost 10 cm of finely laminated sapropel at the initiation of the PETM occurs where %TOC, C_org_/P_tot_, C_org_/Al_tot_ (Fig. 2), trace metal enrichments and lycopane/*n*-C35 alkane indices are the highest, suggesting severe anoxia. Fe-speciation data^13^ are also consistent with anoxic and euxinic conditions during the later stages of sapropel deposition when δ^15^N values are also lower than −2‰ (Fig. 2).

The paleoceanographic mechanism for the overall ^15^N-depletion is consistent with a shift in the style of circulation during the PETM in the Eastern Peri-Tethys. Transgression, intensification of the hydrologic cycle and an increased flux of fresh water from Eurasia delivered weathering-derived nutrients and maintained an estuarine style of circulation. It should be noted that this mechanism does not require substantial reductions in surface water salinities, which are not supported by the microfossil record^15^. Rather, the dynamical response of relatively enclosed bodies of water to an increased freshwater flux from land can induce estuarine circulation with relatively small variations in salinities^31^. The net effect of circulation changes during the PETM in the Tethys enhanced nutrient trapping, increased productivity, and a led to a shallow chemocline^20^ (Fig. 4). Throughout the PETM, a significant relationship between C_org_/P_tot_ and C_org_/Al_tot_ with δ^15^N (Supplementary Fig. 1) suggests that nutrient recycling played a fundamental role in maintaining productivity, anoxia, and the flux of ammonium from deeper waters. Additionally, higher phosphate availability may have enhanced nitrogen fixation, to some degree, lowering the overall δ^15^N of DIN.

Following the initial deposition of the sapropel level, with δ^15^N of −3.2‰, δ^15^N increased to +0.5‰. Coincident with ^15^N-enrichment, Fe-speciation indicates more oxidizing conditions, and %TOC, C_org_/P_tot_, and C_org_/Al_tot_ decrease. At least episodically oxidizing conditions at the benthic boundary layer are indicated by the occurrence of *Chondrites* burrows^15,54^ and a lack of sedimentary laminae. Collectively, these data suggest that the stratification and euxinia at the initiation of the PETM waned, allowing ventilation of the benthic boundary layer. A potential mechanism for this transition may lie in terrestrial sequences of the Pyrenees^29^ where fluvial sedimentology demonstrates a clear shift toward significantly higher discharges with extreme seasonality in the early stages of the PETM, but this effect is not persistent. If enhanced riverine nutrient flux, estuarine circulation, and relative sea level variations^12,13,15^ were the primary controls of sapropel development, as we suggest, a similar climatic regime apparently operated in the region of Kheu River in the Eastern Peri-Tethys. The initial stage of enhanced stratification, anoxia, and ^15^N-depletion was coincident with the rapid decrease in δ^13^C at the onset of the PETM (Fig. 2). The later interval of ^15^N-depletion and renewed anoxia that occurred near the termination of the plateau phase of the PETM likely has a similar mechanism: an enhanced riverine flux that maintained stratification and provided nutrients (Fig. 2). Hydroclimate perturbations at the initiation of the PETM are well-known^29^, but the later reinvigoration of anoxia at Kheu River may be linked to regional fluctuations in relative sea-level and weathering^15^.

The interval of reduced stratification during the middle of the PETM at Kheu River would have increased the volume of low-O_2_ hypoxic or suboxic waters at the expense of anoxic water (hypoxia is where O_2_ concentrations are between 1 and 30% saturation and suboxia where O_2_ < 1% saturation)^55^. A transition from fully anoxic conditions to suboxic conditions would have stimulated the activity of dissimilatory nitrogen redox metabolisms; ammonia oxidation (nitrification), nitrate/nitrite reduction, and anammox^56^. The increase in δ^15^N that occurred over this interval of time may signal the ^15^N-enrichment of DIN that can accompany these processes. While the magnitude of the δ^15^N excursion is equivalent to the increase in denitrification related to post-Pleistocene warming^38^, a δ^15^N of +0.5‰ is not typically considered to be indicative of water column denitrification and may only reflect a reduction in the utilization of ammonium by phytoplankton and low δ^15^N of DIN sourced primarily by diazotrophs.

At Kheu River and elsewhere in the Eastern Peri-Tethys, the development of hypoxic or suboxic conditions during the PETM had the potential to induce positive feedbacks on a rapidly warming world. Nitrous oxide is one of the products of denitrification and ammonia oxidation by bacteria^57^ and archaea^58^ and is a potent greenhouse gas. In the modern ocean at least one-third of the global nitrous oxide flux to the atmosphere is sourced from oxygen-deficient marine waters^59^, and hypoxic and suboxic conditions in shallow shelf sequences are hot spots for nitrous oxide production and its escape to the atmosphere^55,60^. Pulses of oxic waters into the anoxic Gotland Basin in the Baltic Sea have been accompanied by significant increases in the local production of nitrous oxide^61^ and a similar system may have operated at Kheu River. The transition from anoxic to hypoxic/suboxic conditions at Kheu River during the PETM may have stimulated nitrous oxide production, and the expansion of oxygen-deficient waters globally^20^ may have had a similar effect. The degree to which nitrous oxide production operated at a level that impacted PETM warming is unknown, but worthy of more detailed future study.

The PETM bears many of the hallmarks that define OAEs^62^. Over the course of the PETM, warming induced a broad expansion of reducing conditions^13,19,20,23^ and our data suggest a significant reorganization of the nitrogen cycle in response to these redox changes at Kheu River. Unlike many Mesozoic OAEs, PETM organic-matter-rich deposition was entirely confined to shelves and relatively enclosed basins. We speculate that this difference is the result of basin geometries and circulation patterns during the PETM that were less well-suited to broader development of organic matter-rich deposition. During some Mesozoic events, such as Oceanic Anoxic Event 2 (~94 Ma), estuarine circulation and nutrient trapping in the Atlantic and Tethys Oceans nurtured black shale deposition on shelves and deep basins over long periods of time^63^. Despite these differences, the Tethys during the PETM had geochemical conditions, including the nitrogen isotope record, which were essentially indistinguishable from Mesozoic OAEs (Fig. 3).

Today, near-shore regions are clearly sensitive to nutrient loading^2^. Coastal anoxia is exacerbated by the direct addition of nutrients from human activities^60^, but the compounding effects of warming over the coming centuries on near-shore regions is hard to predict. For this reason we look to the geologic record as an analogue, for which the PETM serves as the most recent example of rapid, CO_2_-driven global warming in a non-glacial climatic state^1^. It is clear that near-shore regions in some parts of the Early Eocene world experienced extreme environmental shifts. Given the fact that the rate of PETM warming was likely significantly slower^8^ than present-day warming, the consequences of anthropogenic climate change on biogeochemical systems may be extreme. This argument underscores the sensitivity of modern coastal ecosystems to the consequences of rapid climate change.

## Methods

### Nitrogen isotope analyses

Nitrogen isotopic analyses of decarbonated rock powders were performed in the Syracuse University GAPP Lab using an Elementar Isotope Cube elemental analyzer (EA) coupled directly to an Isoprime 100 isotope ratio mass spectrometer (IRMS) when using conventional techniques for EA-IRMS. EA conditions were as follows: helium purge was set for 45 s, oxidation and reduction reactor temperatures were 1100 °C and 650 °C, respectively; helium carrier gas flow was 230 ml/min; and the O_2_ pulse was set for 90 s at a flow rate of 25 ml/min. Reproducibility for replicate samples, in-house standard materials and international reference materials (IAEA N1 ammonium sulfate [+0.4‰], N2 ammonium sulfate [+20.3‰], NIST 1547 peach leaves [+2.0‰]; and Messel Oil Shale [+7.0‰]) was often better than ±0.15‰, but are reported as ±0.2‰ to reflect the known nitrogen isotopic composition of reference materials relative to atmospheric N_2_ [0‰].

Conventional nitrogen isotopic analyses of organic-matter-rich sediments with high C/N can prove difficult. Sample CO_2_ carryover into subsequent samples is the most significant concern and can generate neoformed CO^+^ within the ion source resulting in significant ^15^N-depletion. Despite the organic richness of these samples, the low thermal maturity allowed for complete combustion indicated by clean blanks and accuracy of interspersed reference materials. As well, the trap and purge system for CO_2_ analysis in the Elementar Isotope EA traps any CO or residual CO_2_ from the previous sample. The possibility of CO generation during the course of sample runs is further achieved by observing *m*/*z* 30 during N_2_ elution which indicates the co-elution of ^12^C^18^O; the presence of the more abundant ^12^C^16^O is masked by at *m*/*z* 28 by N_2_ but can the cause of anomalous ^15^N-depletion.

### Nitrogen isotope analyses employing nanoEA

Alternative methods for analysis of the nitrogen isotopic composition of Kheu Rivers samples was employed for samples where the available material was extremely limited, or where organic carbon content was below 0.5 wt%. The “nanoEA” method that we employed was similar to that described in Polissar et al.^64^. The nanoEA comprises an Elementar Isotope Cube EA coupled to an Isoprime Trace Gas analyzer. The Trace Gas is used for N_2_ trapping and chromatographic focusing prior to sample gas introduction into the Isoprime 100 stable isotope mass spectrometer. Sample powders were loaded into tin capsules, evacuated, and purged with argon prior to introduction into the EA to remove interstitial atmospheric N_2_. EA conditions were as follows: helium purge was set for 45 s, oxidation and reduction reactor temperatures were 1100 and 650 °C, respectively; helium carrier gas flow was 150 ml/min; and the O_2_ pulse was set for 90 s. During sample analyses, the full flow of the EA is diverted to an automated silica gel-filled cryotrap that is immersed in liquid nitrogen over the duration that N_2_ gas is generated during sample combustion. The N_2_ trap is switched to a low-flow He carrier gas (2 ml/min) via an automated Vici 6-port Valco valve and released to the IRMS through an Agilent CarboBond column (25 m × 0.53 mm × 5 µm).

NanoEA Samples were run in triplicate using sequentially larger samples (i.e., 2, 4, and 6 mg) and blank corrected using Keeling-style plots. International reference materials IAEA N1 ammonium sulfate [+0.4‰], N2 ammonium sulfate [+20.3‰], NIST 1547 peach leaves [+2.0‰]; and Messel Oil Shale [+7.0‰] were run in a similar manner, and in quantities of N that bracketed the N-content of the sample materials. The resulting blank corrected sample and standard data were corrected to accepted values for the reference materials using the correction scheme described in Coplen et al.^65^. Reproducibility for samples and standards using nanoEA is ±0.25‰, and approaches the reported nitrogen isotopic composition of the reference materials (±0.2‰). Standard EA-IRMS techniques reproducibility was better than ±0.2‰.

### Data availability

All data considered in the manuscript are provided in the Supplementary Information that accompanies this manuscript.

## Electronic supplementary material

Supplementary Information
