# Supplementary material for: Perturbation to the nitrogen cycle during rapid Early Eocene global warming
Source: Nat Commun. 2018 Aug 9;9:3186. doi: 10.1038/s41467-018-05486-w (PMC6085358; doi:10.1038/s41467-018-05486-w)
Supplement: Supplementary file 1 — Supplementary Information [file 41467_2018_5486_MOESM1_ESM.pdf]

Supplementary Information

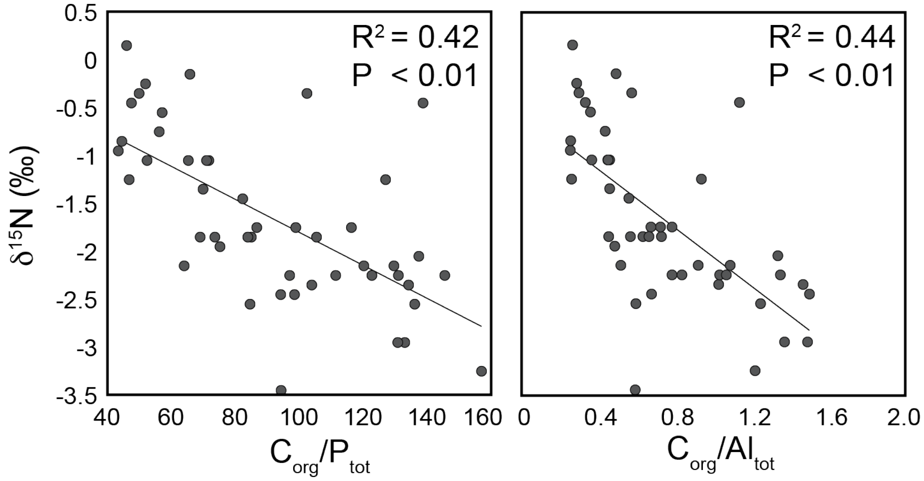

Supplementary Fig. 1. Cross plots of C_{org}/P_{tot} and C_{org}/Al_{tot} versus $\delta^{15}N$.

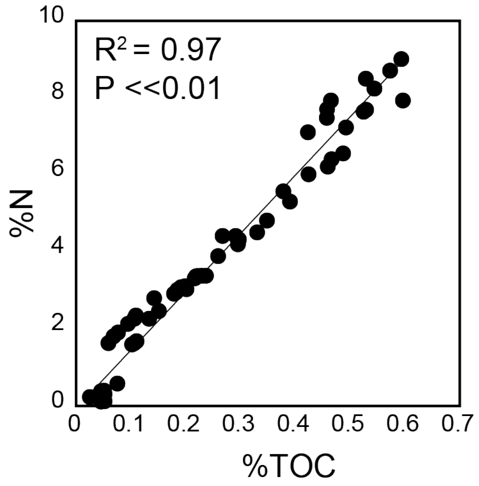

Supplementary Fig. 2. Cross plots of wt. %N and wt % TOC.

Supplementary Table 1. Nitrogen contents and nitrogen isotope data are new to this work, additional data were previously reported in Dickson et al.[1]

Outcrop Level	$\delta^{13}C$	wt.% TOC	wt.% N	C/N	$\delta^{15}N$	Fe_{HR}/Fe_{tot}	C_{org}/P_{tot}	C_{org}/Al_{tot}	Lithology
313	-26.2	0.13					15	0.04	siliceous mudstone
298	-25.7	0.21					12	0.04	siliceous mudstone
283	-26.3	0.17	0.03	6.8	4.1		25	0.06	siliceous mudstone
268	-25.7	0.16					14	0.02	siliceous mudstone
253	-26.1	0.26					22	0.04	siliceous mudstone
238	-25.8	0.32	0.05	7.5	5.4		27	0.06	calcareous mudstone
223	-25.9	0.28					21	0.04	calcareous mudstone
208	-25.7	0.34	0.06	7.2	4.2		25	0.06	calcareous mudstone
193	-26	0.21					12	0.04	calcareous mudstone
178	-26.2	0.22	0.05		2	0.07	14	0.04	calcareous mudstone
163	-26.2	0.23				0.06	15	0.04	calcareous mudstone
148	-26.4	0.26	0.05	5.9	4.5		17	0.04	calcareous mudstone
133	-26.3	0.22			6.6	0.1	14	0.04	calcareous mudstone
118	-27.1	0.23	0.05	4.9	4.3		11	0.04	calcareous mudstone
103	-27	0.27			4.4	0.1	12	0.04	calcareous mudstone
88	-27.8	0.24	0.06	4.8	3.4		7	0.04	calcareous mudstone
73	-28.8	0.52	0.08	7.5	1.7	0.09	14	0.08	calcareous mudstone
56.8	-29.6	1.74	0.07	27.8	-0.2		51	0.28	organic matter-rich mudstone
55.7	-29.6	1.84	0.08	26.4	-0.3		49	0.28	organic matter-rich mudstone
54.6	-29.8	1.57	0.06	28.7	-1.2	0.3	46	0.24	organic matter-rich mudstone
52.6	-29.6	2.2	0.11	23	-0.5	0.32	57	0.34	organic matter-rich mudstone
51.1	-29.9	2.73	0.15	21.7	-0.7		56	0.42	organic matter-rich mudstone
50.45	-29.9	7.17	0.5	16.8	-2.3	0.23	133	1.02	organic matter-rich mudstone
48.8	-29.7	2.28	0.11	23.3	-1		52	0.36	organic matter-rich mudstone
50.45	-29.9	7.62	0.53	16.7	-2.1		119	1.08	organic matter-rich mudstone
48.8	-29.8	2.07	0.1	24.3	-0.4		47	0.32	organic matter-rich mudstone
47.8	-32.7	7.87	0.6	15.3	-0.4	0.31	137	1.12	organic matter-rich mudstone
47.3	-30.2				-2	0.3			organic matter-rich mudstone
46.8	-29.9	6.49	0.49	15.4	-2.1	0.35	128	0.9	organic matter-rich mudstone
46.3	-30	4.44	0.34	15.5	-2.4		97	0.66	organic matter-rich mudstone
44.2	-29.9	3.32	0.24	16	-2.1		63	0.5	organic matter-rich mudstone
44.2	-29.9				-1.8				organic matter-rich mudstone

42.3	-29.9	4.75	0.35	15.7	-1.8	0.33	104	0.72	organic matter-rich mudstone
40.8	-29.9	6.34	0.47	15.7	-2.2	0.4	129	1.02	organic matter-rich mudstone
40.2	-30.1				-0.4				organic matter-rich mudstone
38.8	-29.9	3.31	0.23	17.1	-3.4	0.53	93	0.58	organic matter-rich mudstone
37.05	-30.2	2.4	0.16	17.9		0.51	53	0.4	organic matter-rich mudstone
35.3	-29.7	1.53	0.11	16.6	-0.9	0.25	43	0.24	organic matter-rich mudstone
32.3	-29.8	5.24	0.4	15.5	-1.7	0.29	115	0.78	organic matter-rich mudstone
30.3	-30.1	3.32	0.23	16.6	-1.8	0.36	73	0.56	organic matter-rich mudstone
28.8	-32.4	6.15	0.46	15.4	-1.2	0.32	125	0.92	organic matter-rich mudstone
27.3	-30.1	4.13	0.3	16.1	-1.7		98	0.72	organic matter-rich mudstone
26.3	-29.8	3.25	0.22	17.1	-0.3	0.43	101	0.56	organic matter-rich mudstone
25.5	-29.7	1.57	0.11	16.6	-0.8		44	0.24	organic matter-rich mudstone
23.8	-29.7	1.62	0.12	16.5	0.2	0.24	46	0.26	organic matter-rich mudstone
22.3	-29.8	2.97	0.21	16.8	-0.1		65	0.48	organic matter-rich mudstone
19.8	-29.9	3.3	0.22	17.2	-1.4	0.3	81	0.54	organic matter-rich mudstone
19.3	-29.9				0.2				organic matter-rich mudstone
18.55	-29.9	3.82	0.26	16.9	-2.5		84	0.58	organic matter-rich mudstone
17.8	-30	3.02	0.2	18	-1.9	0.26	74	0.48	organic matter-rich mudstone
17.2	-29.8	2.88	0.19	18	-1	0.31	71	0.44	organic matter-rich mudstone
16.6	-30.2	2.95	0.19	18.1	-1		65	0.44	organic matter-rich mudstone
15.65	-30	3	0.2	17.7	-1.8	0.29	68	0.44	organic matter-rich mudstone
14.7	-30.1				-3.1				organic matter-rich mudstone
14	-30.1	5.51	0.38	16.8	-2.2	0.26	96	0.78	organic matter-rich mudstone
13.5	-29.8				-0.8				organic matter-rich mudstone
12.75	-30	4.26	0.3	16.5	-1.8	0.2	84	0.62	organic matter-rich mudstone
12	-30.2				-1.7				organic matter-rich mudstone
11	-29.8	2.85	0.18	18.1	-1	0.34	70	0.44	organic matter-rich mudstone
10	-29.8	3.04	0.2	17.4	-1.3	0.3	69	0.44	organic matter-rich mudstone with laminations
9.5	-29.8				-2				organic matter-rich mudstone with laminations

9	-30.1	4.35	0.3	17.2	-1.7		86	0.66	organic matter-rich mudstone with laminations
8	-30.3	5.95	0.43	16.2	-2.2	0.32	110	0.82	organic matter-rich mudstone with laminations
7.5	-30.3				-1.9				organic matter-rich mudstone with laminations
7	-30.3	7.58	0.53	16.7	-2.2	0.35	121	1.06	organic matter-rich mudstone with laminations
6.5	-30.3				-2.8				organic matter-rich mudstone with laminations
6	-30.4	8.64	0.58	17.5	-3.2	0.38	155	1.2	organic matter-rich mudstone with laminations
5.33	-30.7	8.43	0.53	18.4	-2.9	0.63	131	1.48	organic matter-rich mudstone with laminations
4.67	-30.6				-2.5				organic matter-rich mudstone with laminations
4	-30.6	7.04	0.43	19.2	-2.2	0.64	144	1.34	organic matter-rich mudstone with laminations
3.5	-30.4	7.42	0.46	18.7	-2.9	0.55	129	1.36	organic matter-rich mudstone with laminations
3	-30.6	7.64	0.46	19.3	-2.3	0.55	103	1.46	organic matter-rich mudstone with laminations
2.25	-30.5	7.87	0.47	19.5	-2.4	0.58	93	1.48	organic matter-rich mudstone with laminations
1.5	-30.5	8.94	0.6	17.4	-2	0.4	136	1.32	organic matter-rich mudstone with laminations
1.2	-31.3	8.18	0.55	17.4	-2.5	0.45	134	1.24	organic matter-rich mudstone with laminations
0.8	-30.2	4.35	0.27	18.7	-1.8	0.38	83	0.66	organic matter-rich mudstone with laminations
0.4	-29.5				-2.6				organic matter-rich mudstone with laminations
0	-29.2	2.2	0.14	18.5			45	0.3	organic matter-rich mudstone with laminations
-15	-25.5	0.15			2.1	0.11	8	0.02	mudstone
-30		0.14	0.05	3.1	2.2	0.09	6	0.02	mudstone
-45	-25.4	0.13				0.08	5	0.02	mudstone
-60	-25.4	0.12			6.2	0.08	6	0.02	calcareous mudstone
-75	-25.4	0.06	0.06	1.2	4.5		3	0.02	calcareous mudstone
-90	-25.4	0.1	0.05		2.1		5	0.02	calcareous mudstone
-105	-25.3	0.15	0.05		4.2		8	0.02	calcareous mudstone

-120	-25.4	0.05	0.05		6	3		marlstone
-135	-25.4	0.05	0.05		3.2	2		marlstone
-150	-25.4	0.05				2		marlstone
-165	-25.2	0.08				4	0.02	marlstone
-180	-25.2	0.06	0.05	1.3	4.8	4	0.02	marlstone
-195	-25.3	0.1	0.05	2.3	4.4	7	0.02	marlstone
-210	-25.2	0.07	0.05		2.2	5	0.02	marlstone

Supplementary References

1. Dickson, A. J., Cohen, A. S., Taylor, K. & Shcherbinina, E. The spread of marine anoxia on the Northern Tethys margin during the Paleocene-Eocene. *Paleoceanography* (2014). doi:10.1002/2014PA002629
